# Proresolving Lipid Mediators: Endogenous Modulators of Oxidative Stress

**DOI:** 10.1155/2019/8107265

**Published:** 2019-06-18

**Authors:** Alessandro Leuti, Mauro Maccarrone, Valerio Chiurchiù

**Affiliations:** ^1^Department of Medicine, Campus Bio-Medico University of Rome, Via Alvaro del Portillo 21, 00128 Rome, Italy; ^2^European Center for Brain Research, IRCCS Santa Lucia Foundation, Via del Fosso di Fiorano 64, 00143 Rome, Italy

## Abstract

Specialized proresolving mediators (SPMs) are a novel class of endogenous lipids, derived by *ω*-6 and *ω*-3 essential polyunsaturated fatty acids such as arachidonic acid (AA), docosahexaenoic acid (DHA), and eicosapentaenoic acid (EPA) that trigger and orchestrate the resolution of inflammation, which is the series of cellular and molecular events that leads to spontaneous regression of inflammatory processes and restoring of tissue homeostasis. These lipids are emerging as highly effective therapeutic agents that exert their immunoregulatory activity by activating the proresolving pathway, as reported by a consistent bulk of evidences gathered in the last two decades since their discovery. The production of reactive oxygen (ROS) and nitrogen (RNS) species by immune cells plays indeed an important role in the inflammatory mechanisms of host defence, and it is now clear that oxidative stress, viewed as an imbalance between such species and their elimination, can lead to many chronic inflammatory diseases. This review, the first of its kind, is aimed at exploring the manifold effects of SPMs on modulation of reactive species production, along with the mechanisms through which they either inhibit molecular signalling pathways that are activated by oxidative stress or induce the expression of endogenous antioxidant systems. Furthermore, the possible role of SPMs in oxidative stress-mediated chronic disorders is also summarized, suggesting not only that their anti-inflammatory and proresolving properties are strictly associated with their antioxidant role but also that these endogenous lipids might be exploited in the treatment of several pathologies in which uncontrolled production of ROS and RNS or impairment of the antioxidant machinery represents a main pathogenetic mechanism.

## 1. The Resolution of Inflammation

Specialized proresolving mediators (SPMs) are a recently characterized yet ever-growing class of bioactive lipids derived from *ω*-3 and *ω*-6 essential polyunsaturated fatty acids (PUFAs), which have been established as the main gears of the chemical machinery controlling the resolution of inflammation. Resolution of inflammation is the spontaneous regression of both acute and chronic inflammatory responses that avoids persistent inflammation and loss of tissue function [1, 2]. As a matter of fact, the discovery and characterization of these molecules, which include, to date, four classes of lipids, namely, lipoxins, resolvins, maresins, and protectins, have revolutionised the concept of resolution (often referred to as catabasis)—once thought as an idle process resulting from mere dissipation of inflammatory sources. Indeed, it became evident that resolution represents an unquestionably active mechanism, meticulously orchestrated by a vast array of mediators, including annexin 1, melanocortins, galectins, somatostatin, adenosine, endocannabinoids, and of course proresolving lipids [3]. The biosynthesis of virtually all the SPMs identified to date is initiated by the enzymatic addition of oxygen to four dietary fatty acids, namely, *ω*-6 arachidonic acid (AA), *ω*-3 eicosapentaenoic acid (EPA), *ω*-3 docosahexaenoic acid (DHA), and *ω*-3 docosapentaenoic acid (DPA), by means of the concerted action of lipoxygenase (LOX) isozymes, cyclooxygenase-2 (COX-2), and, to a minor extent, cytochrome P450 [1–3]. The genesis of resolution programs represents a sort of paradox, as it relies on the coordinated action of several SPMs that are produced, either directly or through intercellular pathways [4], by the same cells that initiate the inflammation in the first place: innate immune cells (neutrophils and monocytes/macrophages), platelets, and hypoxic endothelial cells. During acute inflammation, these cells use cell *ω*-6 PUFAs, mostly AA that is esterified to cell membrane phospholipids, as the main source to synthesize proinflammatory lipids (like prostanoids, leukotrienes, and thromboxanes). Catabasis is eventually initiated as immune cells undergo a lipid mediator class switch [5], during which two main events occur: (i) AA metabolism is channelled into the production of lipoxins (LXs) that in turn are thought to initiate resolution and (ii) other PUFAs such as EPA, DHA, and DPA start being used as biosynthetic precursors for the other proresolving lipids such as resolvins, protectins, and maresins. As a result, the tissue environment acquires a *ω*-3-based lipid fingerprint, as the inflammatory network enriches with SPMs [6]. SPMs are produced at different times and amounts, with some of them appearing already at early phases of inflammation (i.e., LXs) or reaching their highest level at the peak of acute inflammation (6–12 h), some being produced at later stages (i.e., RvD3) while others remaining at elevated levels from the beginning to the end [2]. The arsenal of SPMs basically acts over time by stopping neutrophil influx at the inflamed site, while promoting local clearance of apoptotic neutrophils and debris through the recruitment of proresolving phenotypes of macrophages, a process that has been termed efferocytosis and that seems to represent a crucial step for catabasis to go on; this, over time, allows tissue regeneration and return to physiological homeostasis [7]. SPMs are bioactive lipids that act by engaging G protein-coupled receptors (GPCRs) that, interestingly, display a rather high level of redundancy with respect to their corresponding ligands. Of note, not only each receptor is often engaged by different SPMs but also a single molecule can act by activating different receptors [8]. During catabasis, SPMs are produced in coordinated waves—with lipoxins appearing earlier and resolvins, protectins, and maresins being produced later [8]; therefore, they act in a time- and cell-dependent manner, through receptors that are differentially expressed mostly by immune and endothelial cells. However, only a handful of SPM-dedicated GPCRs has been discovered to date and the characterization of the full extent of receptors by means of which these lipids control tissue homeostasis represents indeed a crucial challenge for the future. Despite their undisputable role in activating proresolution mechanisms by reducing the cardinal signs of inflammation and promoting the cardinal signs of resolution, it is now clear that SPMs also modulate oxidative stress. This is of no surprise, since production of reactive species and their biological activity are key hallmarks of immune responses.

## 2. Reactive Species and Oxidative Stress

Reactive oxygen and nitrogen species (ROS and RNS, respectively) represent a rather vast class of small oxygen- or nitrogen-containing molecules with particular intrinsic chemical properties—often represented by the presence of one or more unpaired electrons in their molecular and/or atomic orbitals—that confer them a high degree of reactivity [9, 10]. As a matter of fact, these molecules represent possibly one of the most fascinating examples of the compromise that aerobic life was forced to make in order to thrive on planet Earth over 2 billion years ago; indeed, even though oxygen and its related reactive species represent highly toxic and mutagenic molecules, their use in the ancestral aerobes must have represented such a relevant evolutionary advantage that these organisms evolved sophisticated antioxidant defence networks to physiologically cope with their concomitant noxious potential [9]. In eukaryotes, ROS and RNS include many radical and nonradical compounds that are normally generated during metabolic and cellular processes. Superoxide radical (^·^O_2_^−^) is the first ROS that is produced and is generated through the enzymatic or nonenzymatic reduction of triplet oxygen (^3^O_2_): NADPH oxidases (NOX) and xanthine oxidase, which act upon antimicrobial oxidative burst and during purine catabolism, respectively, are the two main sources of enzymatically generated ^·^O_2_^−^, while the nonenzymatic species is mostly produced by leakages in the mitochondrial electron transport chain [11]. This reactive species can be rapidly disproportionated into H_2_O_2_. Additionally, transition metals that are released during inflammatory conditions can act as catalysts in the Haber-Weiss reaction, where H_2_O_2_ and ^·^O_2_^−^ react to generate hydroxyl radicals (^·^OH) [12]. NADPH-dependent oxidation of the guanidine nitrogen of L-arginine, catalyzed by nitric oxide synthase (NOS) isoenzymes, leads to the production of the prototypic RNS, nitric oxide (NO^·^) [11]: this compound is involved in pivotal functions of tissue and cellular homeostasis (e.g., regulation of the vascular tone), and during acute inflammation, it reacts with superoxide to produce peroxynitrite (ONOO^−^), a nonradical RNS that represents a pivotal part of the arsenal unleashed upon invading microbes by neutrophils during immune acute responses and that can be also produced enzymatically through NOX2 and NOS enzymes in phagocytic cells such as neutrophils and macrophages [13]. Unsurprisingly, the major producers of ROS and RNS are indeed immune cells and specifically phagocytic cells (i.e., neutrophils, monocytes/macrophages, and microglia), due to their elevated expression of NADPH oxidase (NOX), NOS, and xanthine oxidase. Even though ROS and RNS mediate many physiological functions and are deeply involved in tissue and cellular homeostasis, from inflammation and immune responses to control of cell signalling and neurotransmission, an excessive and persistent production causes massive lipid peroxidation [14], protein oxidation, and nitrosylation [15] as well as permanent damage to DNA [16], leading to the generation of several toxic biomolecule oxidation products (Table 1).

However, cells are equipped with enzymatic and nonenzymatic antioxidant systems to reduce or eliminate ROS and RNS, thus maintaining redox homeostasis. Antioxidants include naturally occurring molecules of high or low molecular weight, as well as nutritional antioxidants, whose action is strictly linked to their bioavailability. Naturally occurring antioxidants are mainly enzymes such as superoxide dismutase (SOD), catalase, glutathione peroxidase/reductase, and peroxiredoxin (PX) or molecules like glutathione, uric acid, pyruvate, amino acids, transferrin, ferritin, and caeruloplasmin. On the other hand, nutritional antioxidants include lipid-soluble antioxidants (*α*-tocopherol, carotenoids, quinones, and some polyphenols) and water-soluble antioxidants (ascorbic acid and some other polyphenols) [10], as shown in Table 2. Oxidative stress is strictly dependent on the balance between the rate of production and the clearance of radicals, and such an altered balance is mainly regulated by two factors. The first, termed nuclear factor-E2-related factor (Nrf2), is a *cis*-acting organizer of the antioxidant defence network that, by translocating into the nucleus, induces the expression of antioxidant proteins and enzymes such as SOD, glutathione peroxidase, and phase-2 detoxification enzymes including heme oxygenase-1 (HO-1) and peroxiredoxin [10]. The second factor is a recently discovered negative regulator of ROS (NRROS) that reduces ROS production from phagocytes during inflammatory responses [17].

Accumulated evidence indicates that oxidative stress plays a major role in the pathogenesis of virtually every chronic inflammatory disease, from cancer and metabolic and gastrointestinal diseases to autoimmune and neurodegenerative diseases [10, 18]. In light of this, research has put a substantial effort in developing pharmacological strategies that target oxidative stress, and in recent times, SPMs are emerging as novel potential therapeutic agents, by virtue of their ability to contain inflammation without exerting immunosuppression, in situations where the hypoxic environment, aberrant leukocyte activity, or physiopathological dysfunctions of antioxidant mechanisms might build the ideal conditions for detrimental production of ROS and RNS.

## 3. AA-Derived SPMs: Lipoxins

Lipoxins (LXs) represent a small group of SPMs that includes only two mediators, namely, LXA_4_ and LXB_4_, both synthesized from AA in the earlier steps of the resolution phase of inflammation [6]. As catabasis is started and brought to completion, the LX main source is represented by eosinophils, macrophages, dendritic cells, and endothelial cells, which produce them through two main biosynthetic pathways: the first involves the action of neutrophils and platelets, expressing 5-LOX and 12-LOX, respectively, and is shared with the leukotriene synthesis pathway; however, the second requires the catalytic activity of endothelial cell-, monocyte-, and eosinophil-derived 15-LOX and leukocyte-derived 5-LOX [19, 20]. Furthermore, aspirin-mediated covalent inactivation of COX-2 leads to the production of 15-(*R*)-hydroxyeicosatetraenoic acid (15-(*R*)-HETE) which, as the epimer of the natural precursor of LXs, 15-(*S*)-HETE, acts as substrate for the 5-LOX-mediated synthesis of epi-LXs [21]. Interestingly, while LTA_4_ is the precursor of all other leukotrienes [22], it is also a direct precursor of lipoxins, which might suggest that, as inflammation advances and LTA_4_ levels rise, it starts feeding LX synthetic pathways, with subsequent initiation of catabasis. To date, the only receptor known to mediate LX effects is formyl peptide receptor 2 (FPR2), also known as ALX, which is engaged by LXA_4_ during resolution; however, to date, no receptors are known for LXB_4_ [8].

**Figure 1:**
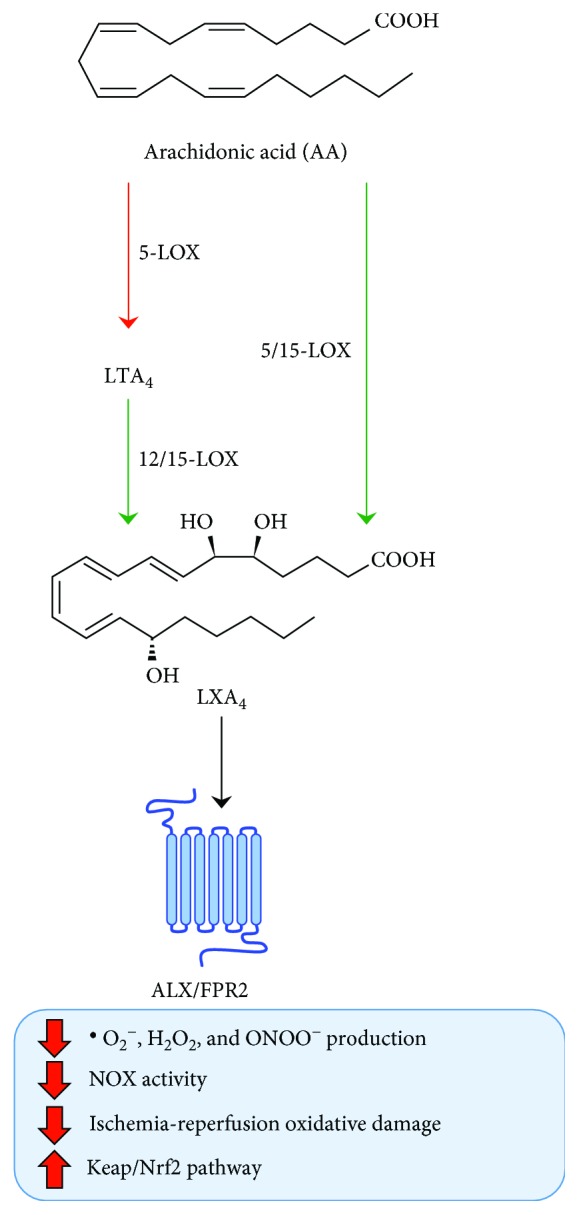
Schematic representation of metabolism of AA-derived SPMs, their receptors, and their functional role in modulation of oxidative stress.

Given the strict chemical relationship between LXs and other AA-derived autacoids that exhibit opposed effects, several authors have investigated the role played by LXs in several pathophysiological processes in which ROS and RNS are involved. In general, LXs and their aspirin-triggered epimers act on reactive species either by interfering with their generation, by remodelling their redox signals, or by inducing antioxidant defences (Figure 1). At the cellular level, the role of LXs in modulating oxidative stress resides, at least in part, in their ability to directly inhibit leukocyte-dependent generation of ^·^O_2_^−^ [23], by inhibiting dephosphorylation of membrane presqualene diphosphate (PSDP), which has been shown to be involved in neutrophil oxidative burst, as well as by inhibiting the assembly of NOX subunits [24, 25]. Since ROS and RNS are produced through a chain of sequential reactions, it is highly plausible that the ability of LXs to hinder the generation of ^·^O_2_^−^ reflects on the production of other related ROS and RNS.

In line with this, LXA_4_ has been demonstrated to reduce production of H_2_O_2_ [26] and ONOO^−^ [27, 28] in primary neutrophils. Interestingly, high levels of ONOO^−^ not only directly induce tissue damage but also induce the expression of key proinflammatory cytokines and chemokines such as IL-6, TNF-*α*, and IL-8 [23, 29]. These cytokines return to control levels when ONOO^−^ pruduction is counteracted production [27] or by LXA_4_ action [6].

On the other hand, the role of LXs in the control of oxidative stress might be way more intricate than expected, in that their by-products, originated through the activity of their two known metabolizing enzymes, 15-hydroxyprostaglandin dehydrogenase (15-PGDH) and leukotriene B_4_ 12-hydroxydehydrogenase (LTB_4_DH), have been demonstrated to inhibit ^·^O_2_^−^ generation in polymorphonucleate cells (PMN) [30], suggesting that both LXs and their by-products are equally involved in the control of redox homeostasis.

In addition, LXs have also been investigated as potential therapeutic agents in the treatment of pathological conditions in which oxidative stress plays a pathogenetic role. Indeed, in ischemia-reperfusion animal models, where the oxidative injury is mostly mediated by impaired blood supply-induced hypoxia or anoxia, LXA_4_ reduced tissue damage in the spinal cord and myocardium [31–33], where its therapeutic effect has been linked to the induction of the Keap/Nrf2-dependent antioxidant response [32, 34]. Interestingly, annexin A1, a distinct nonlipid FPR2/ALX ligand, is involved in the induction of iNOS [35–37], which further strengthens the role of the FPR2 transduction axis in regulating the cellular redox state.

However, the link between AA-derived SPMs and reactive species goes way beyond their ability to avoid collateral damage by simply reducing the levels of harmful ROS and RNS: indeed, the anti-inflammatory properties of aspirin, which also reside in its ability to induce the synthesis of SPM epimers through COX-2 acetylation, are also exerted through the intrinsic ability of AT-LXs to trigger local NO synthesis [38–40], a potent inhibitor of neutrophil chemotaxis and diapedesis [41]. This is further corroborated by the evidence that aspirin is unable to suppress inflammation in mice deficient for endothelial and inducible NOS enzymes [39]. Of note, several studies either linked a dysfunctional LX-FPR2 axis to the pathogenesis of many conditions or reported a therapeutic effect of LX administration on many different diseases that are critically associated with oxidative stress, including Alzheimer's disease (AD) [42], arthritis [43], asthma [44], diabetes [45], atherosclerosis [46], and inflammatory bowel diseases (IBD) [47].

## 4. EPA-Derived SPMs: E-Series Resolvins

Biosynthesis of E-series resolvins (RvE) is initiated by the insertion of molecular oxygen in their essential fatty acid precursor, EPA. This process is usually performed by hypoxic endothelial cells via aspirin-dependent or independent pathways [48]. These routes both start with the synthesis of 18(*R*)-hydroperoxyeicosapentaenoic acid (18(*R*)-HpEPE) that can be catalyzed either by acetylated COX-2 or by cytochrome P450 [1]. Of note, both mammalian and bacterial cytochrome P450 can catalyze this reaction [49]. Eventually, 18(*R*)-HpEPE is either directly transformed into RvE3 by the action of 12/15-LOX [50] or directly converted into RvE1 and RvE2 via a multistep pathway which involves the action of neutrophil-derived 5-LOX in peripheral blood [48, 51]. The only two receptors known to engage E-series resolvins are chemerin receptor 23 (ChemR23) (also known as chemokine-like receptor 1 (CMKLR1)) and BLT1 [52, 53]. The proresolving actions of RvE1 and RvE2 are due to their ability to activate ChemR23 and to antagonize BLT1, which usually mediates the proinflammatory actions of LTB_4_ [8]. To date, the receptor for RvE3 remains to be identified.

**Figure 2:**
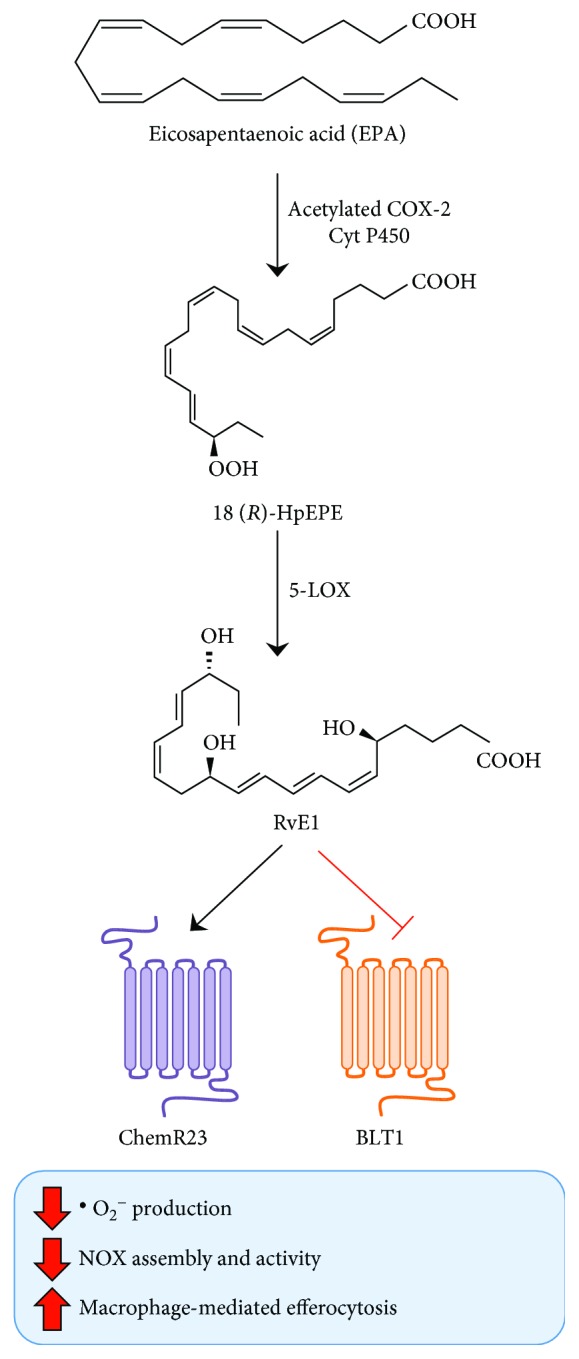
Schematic representation of metabolism of EPA-derived SPMs, their receptors, and their functional role in modulation of oxidative stress.

The literature on the involvement of E-series resolvins in the redox state and oxidative stress includes only very few studies. For instance, in a model of periodontitis, RvE1 was found able to directly suppress ^·^O_2_^−^ production in primary neutrophils, which are otherwise refractory to other SPMs in such paradigm [52, 53]. In a more recent work, RvE1 was reported to reverse cigarette smoke-mediated impairment of efferocytosis in human macrophages, a process that is mainly mediated by their overexposure to ^·^O_2_^−^ in the lungs. This effect apparently resides in RvE1 ability to block membrane translocation of neutrophil cytosolic factor 1 (also known as p47phox), which in turn acts as the organizer of NOX2 [54]. Of note, E-series resolvins might exert a distinct action on different organs, since the reduction of EPA and EPA-derived SPM tissue levels, elicited through the administration of PUFA competitors for the *sn*-2 position in membrane phospholipids, did not result in increased oxidative stress in the kidneys of aged rats [55]. The biosynthetic routes of RvEs, their receptors, and their role in oxidative stress are summarized in Figure 2.

## 5. DHA-Derived SPMs: D-Series Resolvins, Protectins, and Maresins

**Figure 3:**
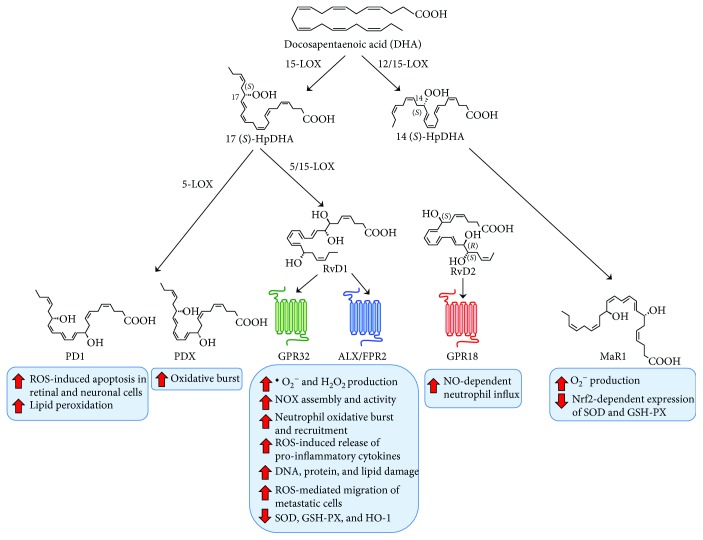
Schematic representation of metabolism of DHA-derived SPMs, their receptors, and their functional role in modulation of oxidative stress.

DHA is a precursor of the most heterogeneous group of SPMs as yet known. This class includes D-resolvins, protectins, and maresins. D-series resolvin synthesis starts off by 15-LOX-mediated conversion of DHA into 17(*S*)-hydroperoxy DHA (17(*S*)-HpDHA). From this, the fate of 17(*S*)-HpDHA depends on 5-LOX action and on which carbon is hydroxylated by its catalytic action. In fact, it can be converted either into 7(*S*)-hydroperoxy-17(*S*)-HDHA to generate RvD1, RvD2, and RvD5 or into 4(*S*)-hydroperoxy-17(*S*)HDHA to generate RvD3, RvD4, and RvD6 [1]. Alternatively, 17(*S*)-HpDHA can be converted to a 16,17-epoxy-docosatriene intermediate that in turn is converted to protectin D1 [56, 57]. Additionally, DHA can be transformed into 14(*S*)-HpDHA by 12-LOX, which, in turn, is converted to maresin 1 and maresin 2 in macrophages [58]. Similarly to E-series resolvins, aspirin-acetylated COX-2 can use the D-resolvin precursor, DHA, to generate 17(*R*)-HpDHA, which is then transformed to epimeric AT-D-resolvins in a 5-LOX-dependent manner [6]. Although molecular targets for maresins and protectins have not been identified yet, three different receptors have been shown to be engaged by almost all D-series resolvins (with the exception of RvD4 and RvD6): (i) FPR2/ALX is engaged, alongside with LXA_4_, by RvD1 and RvD3; (ii) GPR32 (also known as DRV1) is engaged by RvD1, RvD3, and RvD5; (iii) GPR18 (also known as DRV2) is the only receptor known to be engaged by RvD2 [8] (Figure 3).

To date, a number of investigations strongly advocate the idea that the proresolving effects displayed by DHA-derived SPMs depend, at least in part, on their ability to regulate cellular and tissue redox states, thus limiting or suppressing oxidative stress. Similarly to all SPMs, D-series resolvins represent powerful regulators of innate immune cells (especially neutrophils and macrophages) during inflammation, mainly by boosting their proresolving features and/or limiting their pathological role. In macrophages, RvD1 has been found to reduce ROS-mediated release of IL-1*β* [59] and to lower NOX activity by abolishing phosphorylation and assembly of p47 and gp91 [60]. However, since ROS are also inducers of macrophage death caused by excessive efferocytosis [61], the RvD1-mediated dampening of oxidative burst could impair such process. Redox-dependent control over acute inflammation is also achieved by RvDs through a tight grip on neutrophil function, as demonstrated by the fact that RvD1 can limit recruitment of proinflammatory granulocytes and injury due to oxidative stress resulting from the administration of lipid peroxidation-derived aldehydes [62] or LPS-induced acute lung injury [63]. On the other hand, RvD2 was shown to regulate neutrophil influx by acting on the capillary venule tone and leukocyte adhesion via modulation of eNOS activity and local NO levels [64].

DHA-derived SPMs have also been reported to elicit protective actions on a very heterogeneous group of ROS- and RNS-mediated disease models: a number of studies investigated the effects of RvD1 and MaR1 in rodent models of acute lung injury, where part of their proresolving activity was elicited either through Nrf2-dependent expression of GSH-PX and SOD [65, 66] or through reducing aberrant production of RNS [67]. These results were corroborated by the evidence that RvD1 reduced DNA and protein nitrosative damage in a model of chronic lung disease (i.e., emphysema), where endogenous RvD1 levels also inversely correlated with disease severity [68]. Interestingly, lower levels of this RvD1 were linked to augmented NOX expression, ^·^O_2_^−^ production, or the presence of lipid peroxidation products also in other disease models, including atherosclerosis [46], aortic rupture [69], and gastric injury [70]. In all of these studies, administration of RvD1 or DHA reduced oxidative stress and ameliorated clinical phenotypes. RvD1 treatment of UV-irradiated mice also reduced skin oxidative stress and inflammation by acting on several prooxidant enzymes and by restoring glutathione depletion [71].

In liver injury, RvD1 exerted protective and antioxidant effects by reducing specific biomolecule oxidation products and mainly by increasing in glutathione levels, SOD activity, and HO-1 expression [72]. Additionally, although only levels of RvD1 were found to be significantly reduced in patients affected by chronic obstructive pulmonary disease, a condition mostly caused by cigarette smoke-induced oxidative stress [10], both RvD1 and RvD2 were reported to attenuate inflammation and promote resolution in cigarette smoke-exposed human macrophages [73]. Of note, AT-RvD1 has also been reported to enhance resolution of hyperoxic acute lung and renal injuries by reducing MPO activity and by activating Nrf2 and its downstream antioxidant genes [74–77] and to abrogate metastatic cell migration in human cancer cells, although the last effect was paradoxically elicited by lowering Nrf2 expression [78]. In the latter study, since the local generation of ROS was unchanged, probably due to a concomitant AT-RvD1-dependent weakening of glucose metabolism, it is plausible that RvD-dependent modulation of redox homeostasis on malignant cells might operate via other collateral pathways.

MaR1 was also able to strongly reduce ^·^O_2_^−^ production and the subsequent tissue damage in experimental models of vascular dysfunction [79, 80], liver injury [81], renal ischemia/reperfusion injury [82], and skin inflammation [83], while maresin-like lipid mediator 14S,21R-dihydroxy-docosahexaenoic acid improved diabetes-impaired prohealing functions of macrophages by reducing hyperglycaemia-induced ROS production [84] as well as modulated the ability of mesenchymal stem cells to influence ROS generation from macrophage under ischemia/reperfusion conditions [85]. MaR1 was also shown to inhibit endoplasmic reticulum stress via regulation of PPARa-mediated production of oxygen-regulated protein ORP150 [86] and to attenuate mitochondrial dysfunction through the ALX/cAMP/ROS pathway in the cecal ligation and puncture mouse model and also in sepsis patients [87].

Protectins, often called neuroprotectins (PD), belong to the last DHA-derived family of SPMs and include only two mediators, namely, PD1 and its stereoisomer, PDX. PD1 represents probably the best studied among all DHA-derived SPMs, due to its ability to resolve oxidative stress-related inflammation, especially in ROS-induced damages of the brain and retina [56, 88]. This phenomenon seems rather obvious given that brain and retina cell membranes are characterized by the highest amount of DHA among all cells [89]. In this scenario, oxidative stress represents a major danger, in that DHA is a primary target of peroxidation, and the decrease of its levels due to oxidative stress not only impairs basal cellular functions (incidentally, DHA is strongly involved in membrane fluidity and GCPR signal transduction) but also leads to inefficient catabasis and, ultimately, visual and cognitive decline [89].

Accordingly, the protective role of PD1 against oxidative stress in the retina is unequivocal, also due to a stereoselective specific binding of this proresolving mediator to retinal pigment epithelial cells [90]. Indeed, several studies have shown that PD1 biosynthesis is augmented in retinal cells upon oxidative stress [91], probably under the regulation of neurotrophins [92], and this view was further proved in 15-LOX-silenced cells that displayed an exacerbated sensitivity to oxidative stress-induced apoptosis, rescued by PD1 [93]. As a matter of fact, under these conditions, PD1 elicits cytoprotection by significantly dampening ROS-mediated damage, apoptosis, and inflammation [56, 94]. Interestingly, these effects were later found to be mediated by dephosphorylation of Bcl-xL [95] and to depend on the activation of PI3K/Akt signalling [96, 97]. Considering that this signalling pathway has been linked to Nrf2 phosphorylation and degradation [98], this finding suggests that the antioxidant effects of PD1 might involve mechanisms that are different from the other SPMs and that involve cellular adaptation and survival in response to oxidative stress. Unsurprisingly, neuroprotectins seem to play an important role in neurodegeneration and Alzheimer's disease [99, 100], where mitochondrial dysfunction, generation of ROS, and PUFA peroxidation represent early sources of disease initiators, able to lead to progressive neuronal apoptosis [89]. On the other hand, a number of independent studies confirmed PD1 efficacy in reducing the oxidative stress in other pathologies, including a cellular model of Parkinson's disease [101], diabetes [102], and stroke [88]; yet, to date, a single investigation demonstrated the effect of PDX on reducing the oxidative burst in neutrophils through the inhibition of NOX and myeloperoxidase (MPO) [103]. The main ROS-induced human pathologies associated with SPM dysfunctions are listed in Table 3.

## 6. Conclusions

Since the discovery of the first resolvins less than 20 years ago and until the identification of the latest SPMs in the last couple of years, these mediators emerged as a wide family of nearly 30 different molecules that all share the role of being potent immunoresolvents, able to reduce inflammation and to maintain tissue homeostasis by orchestrating manifold body defences and healing processes. However, along and consistent with their proresolving nature, SPMs act also as strong attenuators of oxidative stress. This is consistent with the protective role played by ROS and RNS against pathogens and apoptosis. Accordingly, over 60 papers have reported that all classes of SPMs, LXs, E-series, and D-series Rvs as well as protectins and maresins protect from oxidative stress mainly not only by reducing ROS and RNS production but also by potentiating several naturally occurring antioxidant defences, including modulation of SOD, HO-1, and Nrf2 expression. Among them, most of the literature data on modulation of oxidative stress is on LXs, D-series Rvs, and PD1, whereas E-series Rvs and maresins remain poorly investigated. The limited knowledge on maresins is certainly due to their more recent discovery, but it is likely that they play a great deal of protective antioxidant effects since they are biosynthesized by macrophages, innate immune cells that are major producers of ROS and RNS. To date, research efforts have only focused on a quite small fraction of the rather vast host of proresolving lipids, many of which have just been recently discovered and include glutathionyl, cysteinylglycinyl, and cisteinyl conjugates of maresins (MCTRs), protectins (PCTRs), and resolvins (RCTRs) [104] as well as DPA-derived 13-series resolvins (also known as resolvins T (RvT)) [105], whose biological activity has only started to be investigated. At the same time, only a few receptors are known to mediate SPM activity and most SPMs still lack a molecular target. Discovering the full array of receptors responsible for coordinating resolution of inflammation will open new avenues of research in further unravelling the role of each lipid mediator in the control of oxidative stress. It also seems plausible that redox signalling and oxidative stress work hand in hand with SPMs during normal tissue homeostasis: indeed, ROS/RNS and SPMs are produced by the same cells, reinforcing the growing theory that inflammation and resolution are basically achieved by resetting the same framework as immune responses need to be confined; moreover, the COX- and LOX-dependent synthesis of eicosanoids intrinsically produces ROS and RNS [106] which, upon the induction of resolution, are counteracted by SPMs; this suggests that SPMs might also play a role in modulating the collateral production of oxidative stress derived from the necessity of autacoid metabolism. Quite interestingly, the metabolic pathways of crucial eicosanoids (e.g., leukotrienes) and earlier SPMs (e.g., lipoxins) are critically entwined. Further investigation of the role played by proresolving lipids in this crosstalk will be required in the future in order to shed light on several pathophysiological processes that might be remained obscure so far due to the relatively young age of this research field.

Of note, given that unbalanced redox signals are strongly associated with the pathogenesis of many diseases, from metabolic to autoimmune and neurodegenerative disorders, the ability of SPMs to protect from oxidative stress responses may represent a novel approach to manage such disorders. Indeed, a scenario involving clinical applications for SPMs, one based on their ability to modulate oxidative stress, seems like a promising therapeutic approach, especially in light of the many clinical trials and in vivo studies that recently identified beneficial effects for ROS- and RNS-dampening strategies in several conditions, including chronic obstructive pulmonary disease [107], multiple sclerosis [108], kidney hypertension [109], critically ill patients [110], and diabetes, and overall longevity [111, 112], with several more still ongoing. Interestingly, most of these oxidative stress-related pathologies are also characterized by dysfunctional SPM production or activity, representing a fascinating scenario worth to be exploited for future therapeutic strategies based on endogenous and safe lipid signals.

## Figures and Tables

**Table 1:** Main endogenous oxygen and nitrogen reactive species.

Oxidant	Chemical class	Electronic configuration	Source
^·^O_2_^−^	ROS	Radical	NOX, xanthine oxidase, mitochondria, self-dismutation
H_2_O_2_	ROS	Nonradical	SOD, self-dismutation
^·^OH	ROS	Radical	Haber-Weiss chemistry
NO^·^	RNS	Radical	NOS
ONOO^−^	RNS	Nonradical	Reaction with ^·^O_2_^−^ and NO^·^, NOS, NOX2

**Table 2:** Main endogenous enzymatic and nonenzymatic antioxidant systems.

Antioxidant	Function	Localization
SOD	Removal of ^·^O_2_^−^	Mitochondria
Catalase	Dismutation of H_2_O_2_	Peroxisomes
Glutathione peroxidase	Removal of H_2_O_2_	Cytoplasm
Glutathione reductase	Reduction of glutathione	Cytoplasm
Peroxiredoxin	Removal of H_2_O_2_, ONOO^−^, hydroperoxides	Cytoplasm, plasma membrane
Transferrin/ferritin/ lactoferrin/ceruloplasmin	Scavenging of transition metals	Plasma and cytoplasm
Glutathione	Scavenging of reactive species	Ubiquitous
Uric acid	Scavenging of reactive species	Ubiquitous
Pyruvate	Scavenging of reactive species	Ubiquitous
Amino acids	Scavenging of reactive species	Ubiquitous

**Table 3:** SPMs in ROS-mediated chronic inflammatory diseases.

SPM	Diseases	References
LXA_4_	Ischemia/reperfusion	[31–33]
LXA_4_	Alzheimer's disease	[42]
LXA_4_	Arthritis	[43]
LXA_4_	Asthma	[44]
LXA_4_	Inflammatory bowel disease	[47]
LXs	Diabetes	[45]
LXs	Cardiovascular diseases	[46]
RvE1	Inflammatory bowel disease	[6]
RvE1	Cardiovascular diseases	[6, 46]
RvD1	COPD and lung ischemia/reperfusion	[10, 65, 67, 68, 75]
RvD2	Lung ischemia/reperfusion	[75]
RvD1	Atherosclerosis	[46]
MaR1	Lung ischemia/reperfusion	[66, 82, 85]
MaR1	Cardiovascular diseases	[46, 79, 80]
MaR1	Diabetes	[84]
PD1	Diabetes	[92]
PD1	Cardiovascular diseases	[46, 78]
PD1	Neurodegenerative diseases	[89–91]
