# Erratum to “Proresolving Lipid Mediators: Endogenous Modulators of Oxidative Stress”

**DOI:** 10.1155/2019/1759464

**Published:** 2019-12-12

**Authors:** Alessandro Leuti, Mauro Maccarrone, Valerio Chiurchiù

**Affiliations:** ^1^Department of Medicine, Campus Bio-Medico University of Rome, Via Alvaro del Portillo 21, 00128 Rome, Italy; ^2^European Center for Brain Research, IRCCS Santa Lucia Foundation, Via del Fosso di Fiorano 64, 00143 Rome, Italy

In the article titled “Proresolving Lipid Mediators: Endogenous Modulators of Oxidative Stress” [1], there were errors in Figure 3 where all the red arrows were in the opposite directions. The corrected version of Figure 3 is shown below:

## Figures and Tables

**Figure 1:**
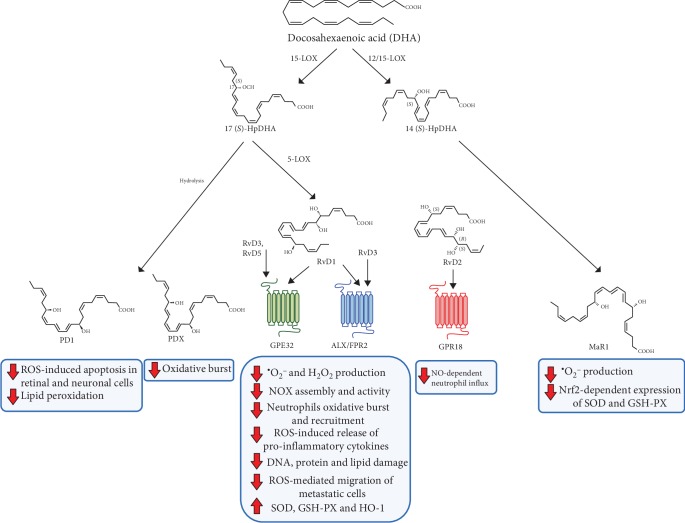
Schematic representation of metabolism of DHA-derived SPMs, their receptors, and their functional role in modulation of oxidative stress.
